# Publisher Correction: Deubiquitinase Usp12 functions noncatalytically to induce autophagy and confer neuroprotection in models of Huntington’s disease

**DOI:** 10.1038/s41467-018-06888-6

**Published:** 2018-10-15

**Authors:** Rebecca Aron, Pasquale Pellegrini, Edward W. Green, Daniel C. Maddison, Kwadwo Opoku-Nsiah, Jinny S. Wong, Aaron C. Daub, Flaviano Giorgini, Steven Finkbeiner

**Affiliations:** 1 Center for Systems and Therapeutics & Taube/Koret Center for Neurodegenerative Disease, Gladstone Institutes, 1650 Owens St., 94158 San Francisco, CA USA; 2 Taube-Koret Center for Neurodegenerative Disease, 94158 San Francisco, CA USA; 3 Department of Genetics and Genome Biology, College of Life Sciences, University of Leicester, Adrian Building, University Road, LE1 7RH Leicester, UK; 4 DKFZ, 69120 Heidelberg, Germany; 5 Graduate Program in Chemistry and Chemical Biology, Department of Pharmaceutical Chemistry, University of California—San Francisco, 94158 San Francisco, CA USA; 6 Graduate Program in Biomedical Sciences, University of California—San Francisco, 94158 San Francisco, CA USA; 7 Graduate Program in Neuroscience, University of California—San Francisco, 94158 San Francisco, CA USA; 8 Graduate Program in Medical Scientist Training Program, University of California—San Francisco, 94158 San Francisco, CA USA; 9 Department of Neurology and Physiology, University of California—San Francisco, 94158 San Francisco, CA USA; 10 Present Address: Yumanity Therapeutics, 02139 Cambridge, MA USA

Correction to: *Nature Communications* 10.1038/s41467-018-05653-z, published online 28 September 2018

The original version of this article incorrectly gave a publication date of 8 October 2018; this should have been 28 September 2018. This has now been corrected in the PDF and HTML versions of the Article.

