# Author Correction: Deubiquitinase Usp12 functions noncatalytically to induce autophagy and confer neuroprotection in models of Huntington’s disease

**DOI:** 10.1038/s41467-020-14582-9

**Published:** 2020-02-21

**Authors:** Rebecca Aron, Pasquale Pellegrini, Edward W. Green, Daniel C. Maddison, Kwadwo Opoku-Nsiah, Ana Osório Oliveira, Jinny S. Wong, Aaron C. Daub, Flaviano Giorgini, Paul Muchowski, Steven Finkbeiner

**Affiliations:** 1 Center for Systems and Therapeutics & Taube/Koret Center for Neurodegenerative Disease, Gladstone Institutes, 1650 Owens St., San Francisco, CA 94158 USA; 2 Taube-Koret Center for Neurodegenerative Disease, San Francisco, CA 94158 USA; 3 Department of Genetics and Genome Biology, College of Life Sciences, University of Leicester, Adrian Building, University Road, Leicester, LE1 7RH UK; 4 DKFZ, Heidelberg, 69120 Germany; 5 Graduate Program in Chemistry and Chemical Biology, Department of Pharmaceutical Chemistry, University of California—San Francisco, San Francisco, CA 94158 USA; 6 Graduate Program in Biomedical Sciences, University of California—San Francisco, San Francisco, CA 94158 USA; 7 Graduate Program in Neuroscience, University of California—San Francisco, San Francisco, CA 94158 USA; 8 Graduate Program in Medical Scientist Training Program, University of California—San Francisco, San Francisco, CA 94158 USA; 9 Department of Neurology and Physiology, University of California—San Francisco, San Francisco, CA 94158 USA; 10 Present Address: Yumanity Therapeutics, Cambridge, MA 02139 USA

**Keywords:** Enzyme mechanisms, Huntington's disease

Correction to: *Nature Communications* 10.1038/s41467-018-05653-z, published online 28 September 2018

The original version of this Article omitted from the author list the 6th and 10th authors, Ana Osório Oliveira and Paul Muchowski, respectively, both at the Gladstone Institute at the time of their participation to the project. The following was added to the Author contributions: ‘A.O.O. provided technical assistance. P.M. contributed to the conceptual inception of the project and the initial stages of its development.’ This has been corrected in both the PDF and HTML versions of the Article.

