# Publisher Correction: Deubiquitinase Usp12 functions noncatalytically to induce autophagy and confer neuroprotection in models of Huntington’s disease

**DOI:** 10.1038/s41467-020-16216-6

**Published:** 2020-05-07

**Authors:** Rebecca Aron, Pasquale Pellegrini, Edward W. Green, Daniel C. Maddison, Kwadwo Opoku-Nsiah, Ana Osório Oliveira, Jinny S. Wong, Aaron C. Daub, Flaviano Giorgini, Paul Muchowski, Steven Finkbeiner

**Affiliations:** 1 Center for Systems and Therapeutics & Taube/Koret Center for Neurodegenerative Disease, Gladstone Institutes, 1650 Owens St., San Francisco, CA 94158 USA; 2 Taube-Koret Center for Neurodegenerative Disease, San Francisco, CA 94158 USA; 3 Department of Genetics and Genome Biology, College of Life Sciences, University of Leicester, Adrian Building, University Road, Leicester, LE1 7RH UK; 4 DKFZ, Heidelberg, 69120 Germany; 5 Graduate Program in Chemistry and Chemical Biology, Department of Pharmaceutical Chemistry, University of California—San Francisco, San Francisco, CA 94158 USA; 6 Graduate Program in Biomedical Sciences, University of California—San Francisco, San Francisco, CA 94158 USA; 7 Graduate Program in Neuroscience, University of California—San Francisco, San Francisco, CA 94158 USA; 8 Graduate Program in Medical Scientist Training Program, University of California—San Francisco, San Francisco, CA 94158 USA; 9 Department of Neurology and Physiology, University of California—San Francisco, San Francisco, CA 94158 USA; 10 Present Address: Yumanity Therapeutics, Cambridge, MA 02139 USA

**Keywords:** Enzyme mechanisms, Huntington's disease

Correction to: *Nature Communications* 10.1038/s41467-020-14582-9, published online 21 February 2020, and 10.1038/s41467-018-05653-z, published online 28 September 2018.

A correction was issued for the original version of this Article which contained an error in the spelling of the author Ana Osório Oliveira, which was incorrectly written as Anna Osorio Oliviera. This has now been corrected in both the PDF and HTML versions of the Article and of the Correction.

