# Supplementary material for: Deubiquitinase Usp12 functions noncatalytically to induce autophagy and confer neuroprotection in models of Huntington’s disease
Source: Nat Commun. 2018 Sep 28;9:3191. doi: 10.1038/s41467-018-05653-z (PMC6162324; doi:10.1038/s41467-018-05653-z)
Supplement: Supplementary file 1 — Supplementary Information [file 41467_2018_5653_MOESM1_ESM.pdf]

# Deubiquitinase Usp12 functions noncatalytically to induce neuronal autophagy and confer neuroprotection in models of Huntington's disease

Aron et al.

## Supplementary Figure 1

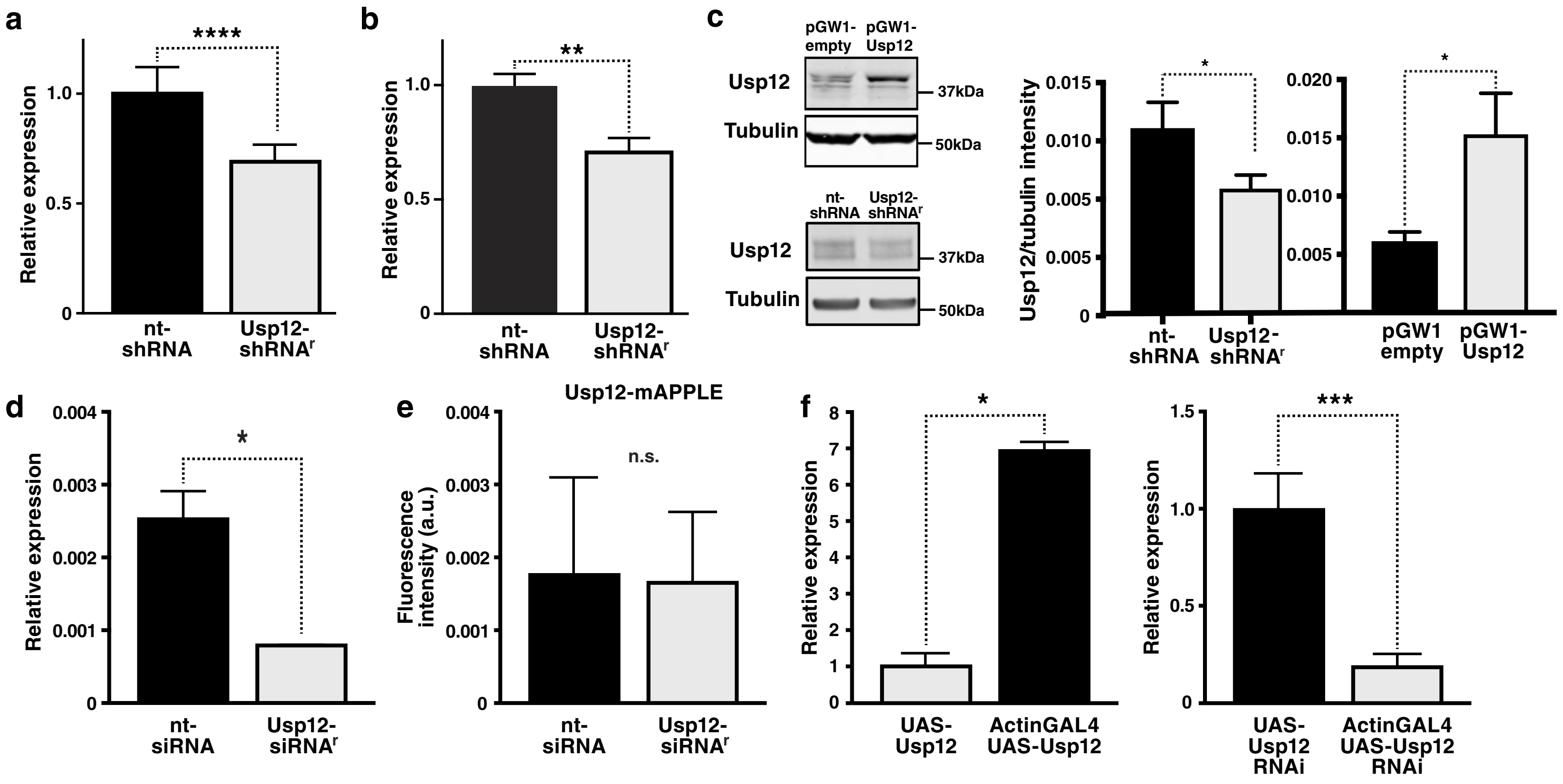


**Supplementary Figure 1.** shRNA-mediated knockdown of Usp12 in PC12 cells and primary neurons. (a) qPCR analysis of Usp12 mRNA levels in rat cell line (PC12) transfected with Usp12-shRNA$^r$ plasmid. With approximately 50% transfection efficiency, as measured by GFP plasmid co-transfection, Usp12 mRNA knockdown averaged 30–50%. Atp5b is used as the reference gene for quantification. Usp12 mRNA levels in Usp12-shRNA$^r$ treated cells are then normalized to Usp12-mRNA levels in cells treated with nt-shRNA. (b) qPCR analysis of Usp12 mRNA levels in primary rodent neurons transduced with Usp12-shRNA$^r$ lentivirus. Due to the low transfection efficiency of primary neurons (<1–5%), we used a lentivirus-mediated shRNA delivery approach to confirm the effectiveness of Usp12-shRNA$^r$ in primary neurons. Primary rat neurons transduced with Usp12-shRNA$^r$ lentivirus show ~30% reduction in Usp12 mRNA levels. (c) Western blot analysis of Usp12 levels of a rat C6 cell line transfected with either the shRNA or the overexpression vector pGW1-Usp12. Graph bars on the right represent the relative intensity of Usp12. (d) Relative Usp12 mRNA levels of a rat C6 cell line transfected with Usp12-siRNA$^r$ (e) Fluorescence intensity of the pGW1-Usp12-mAPPLE plasmid co-transfected with the Usp12-siRNA$^r$ or non-targeting siRNA (p=0.66). (f) Relative mRNA levels of Usp12 overexpression and RNAi knock-down in control fruit flies, driven with the actin-GAL4 driver (see Methods). *p<0.01**p<0.001, ****p<0.0001. Error bars represent s.e.m.

## Supplementary Figure 2

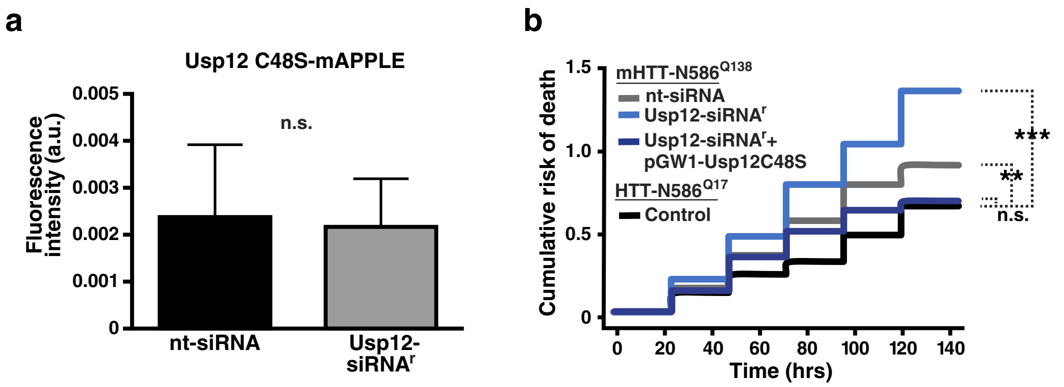


**Supplementary Figure 2**. The catalytic dead Usp12-C48S rescues the Usp12-siRNA$^{r}$ mediated toxicity. (a) Fluorescence intensity of the pGW1-Usp12-C48S-mAPPLE plasmid co-transfected with the Usp12 siRNA$^{r}$ or non-targeting siRNA ($p=0.29$). (b) Cumulative risk of death of the mHttQ138 when co-transfected with either control siRNA, Usp12-siRNA$^{r}$ or Usp12-siRNA$^{r}$ + pGW1-Usp12(C48S) *$p<0.01$**$p<0.001$, ****$p<0.0001$, n.s.=not significant. Statistical information is summarized in Table 3.

## Supplementary Figure 3

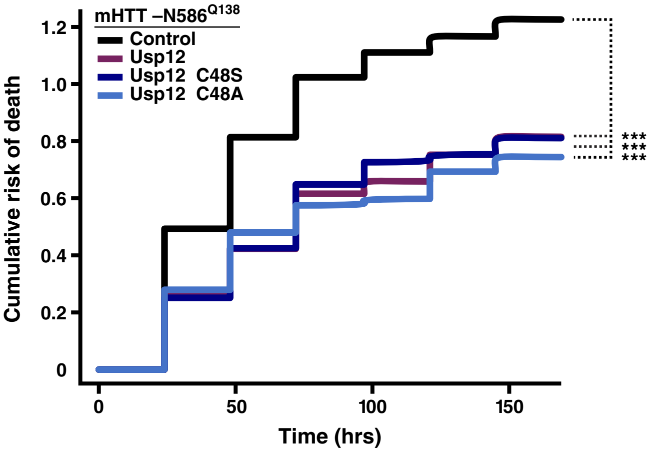


**Supplementary Figure 3.** The active-site cysteine mutant Usp12-C48A suppresses mHTT-N586Q138 toxicity similar to Usp12-C48S. Cumulative risk of death plot for primary neurons co-transfected with mHTT-N568Q138 and empty vector, Usp12, Usp12-C48S, or Usps12-C48A. mHTT-N568Q138 + empty vector vs. Usp12: HR=0.6, 95% CI (0.4750- 0.8134), p=0.000531; vs. Usp12-C48S: HR=0.6, 95% CI (0.4704–0.8222), p=0.000854; vs. Usp12-C48A: HR=0.6, 95% CI (0.4446–0.7788), p=0.000208). Number of neurons per group: mHTT-N568Q138 + empty vector, n=167; mHtt-N568Q138 + Usp12, n=174; mHTT-N568Q138 + Usp12-C48S, n=153; mHtt-N568Q138 + Usp12-C48A, n=160. ***p<0.001.

## Supplementary Figure 4

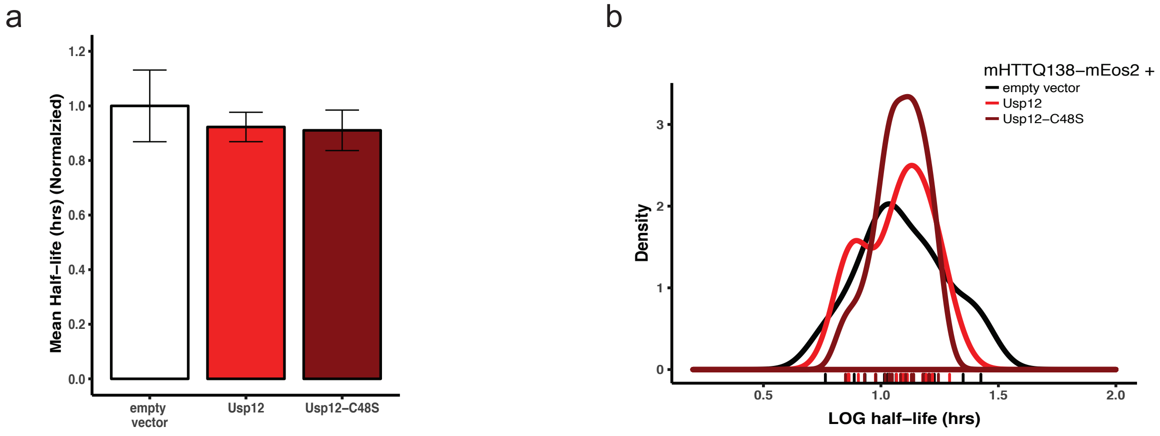


**Supplementary Figure 4.** Usp12 overexpression does not significantly affect the half-life of soluble mHTT in rat primary cortical neurons. Optical pulse-labeling was used to measure the effect of Usp12 or Usp12-C48S on half-life of mHTT-N586Q138-mEos2, similar to the assay described for LC3 half-life (Fig. 6). To measure the effect on only soluble/diffuse mHTT protein, neurons with visible inclusion bodies were excluded from the analysis. (a) Mean half-life of photoconverted mHTT-N586Q138-mEos2 in neurons co-expressing either empty vector, Usp12, or Usp12-C48S. (b) Half-life distributions of individual neurons from (a). Number of neurons per group for representative experiments (n): mHTT-N586Q138+empty vector, n=16; mHTT-N586Q138+Usp12, n=20, mHTT-N586Q138+Usp12-C48S, n=20. Error bars represent s.e.m. Statistical significance of means and distribution determined by Mann-Whitney and Kolmogorov-Smirnov tests, respectively.

## Supplementary Figure 5

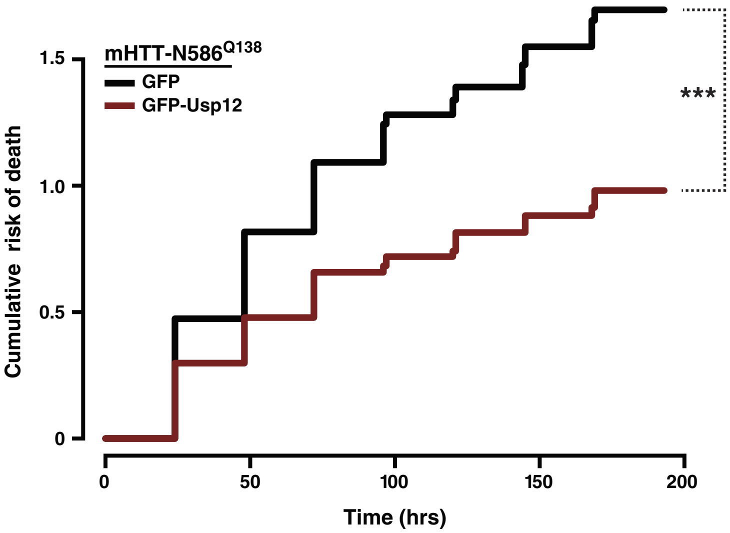


**Supplementary Figure 5.** Fluorescent protein-tagged Usp12 suppresses mHTT toxicity similar to untagged Usp12. Cumulative risk of death plot for primary neurons co-transfected with mHTT-N568Q138 and empty vector or GFP-Usp12. Two experiments combined. Relative risk of death: mHTT-N568Q138 + empty vector vs. mHTT-N568Q138 + GFP-Usp12, HR=0.6, 95% CI (0.494–0.6936), p=5.85e-10. Number of neurons per group: mHTT-N568Q138 + empty vector, n=381, mHTT-N568Q138 + GFP-Usp12, n=384. ***p<0.001

## Supplementary Figure 6

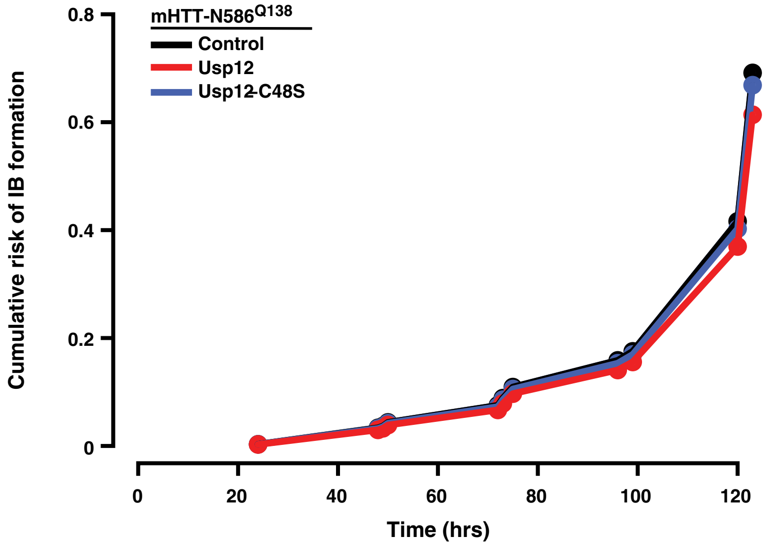


**Supplementary Figure 6.** Cumulative risk of inclusion body formation in neurons expressing mHTT-N586-Q138 and either empty vector, Usp12, or Usp12-C48S. Results are from three experiments combined. Number of neurons per group: mHtt-N568Q138 + empty vector, n=376, mHTT-N568Q138 + Usp12, n=283, mHtt-N568Q138 + Usp12-C48S, n=332.

**Supplementary Figure 7**

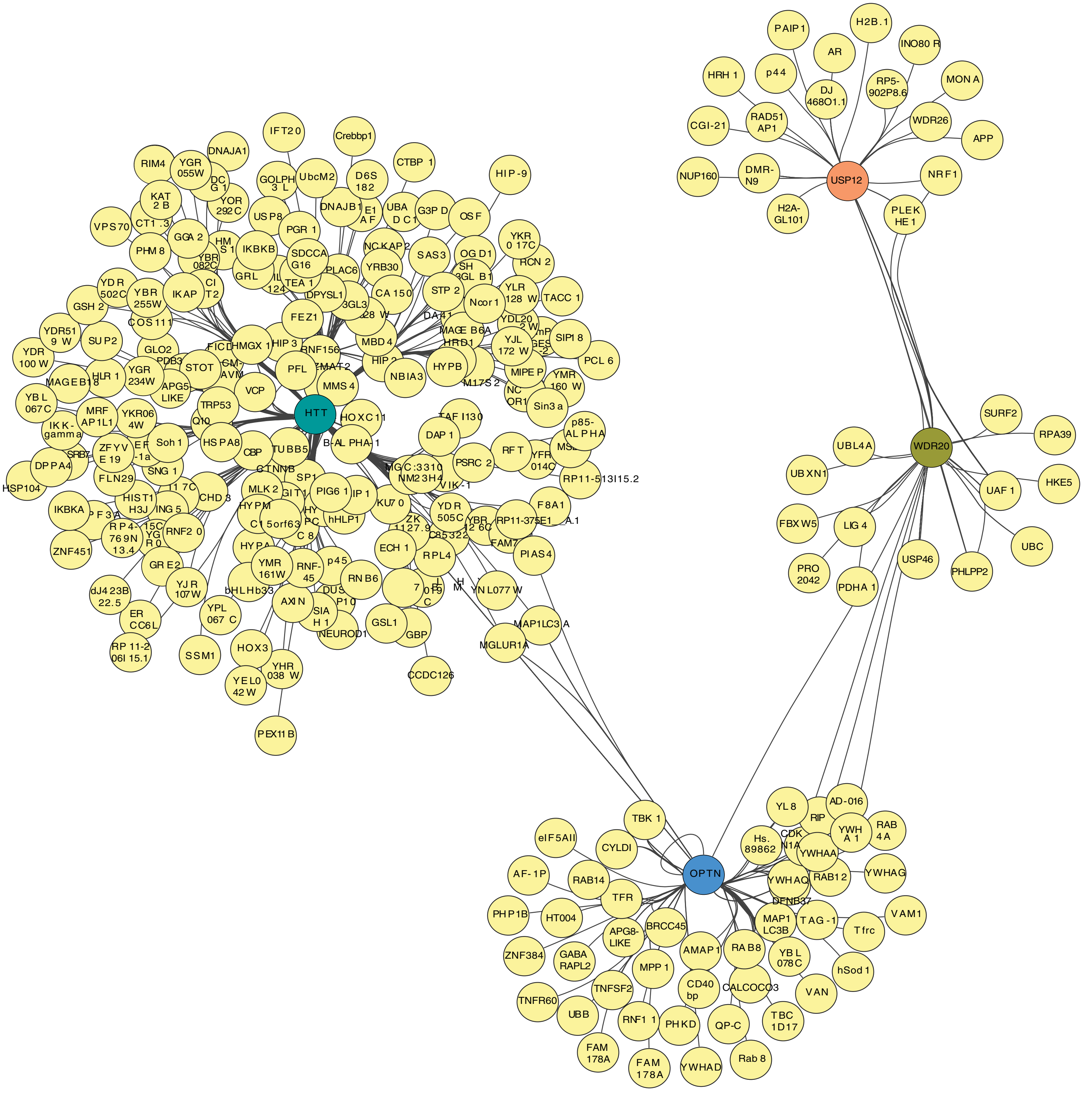


**Supplementary Figure 7.** Detailed map of experimentally validated protein-protein interactions linking Usp12 with the autophagy receptors Optineurin (Optn) and mHTT, based on data collection in BioGRID. Further information is available at www.thebiogrid.com.

Supplementary Figure 8

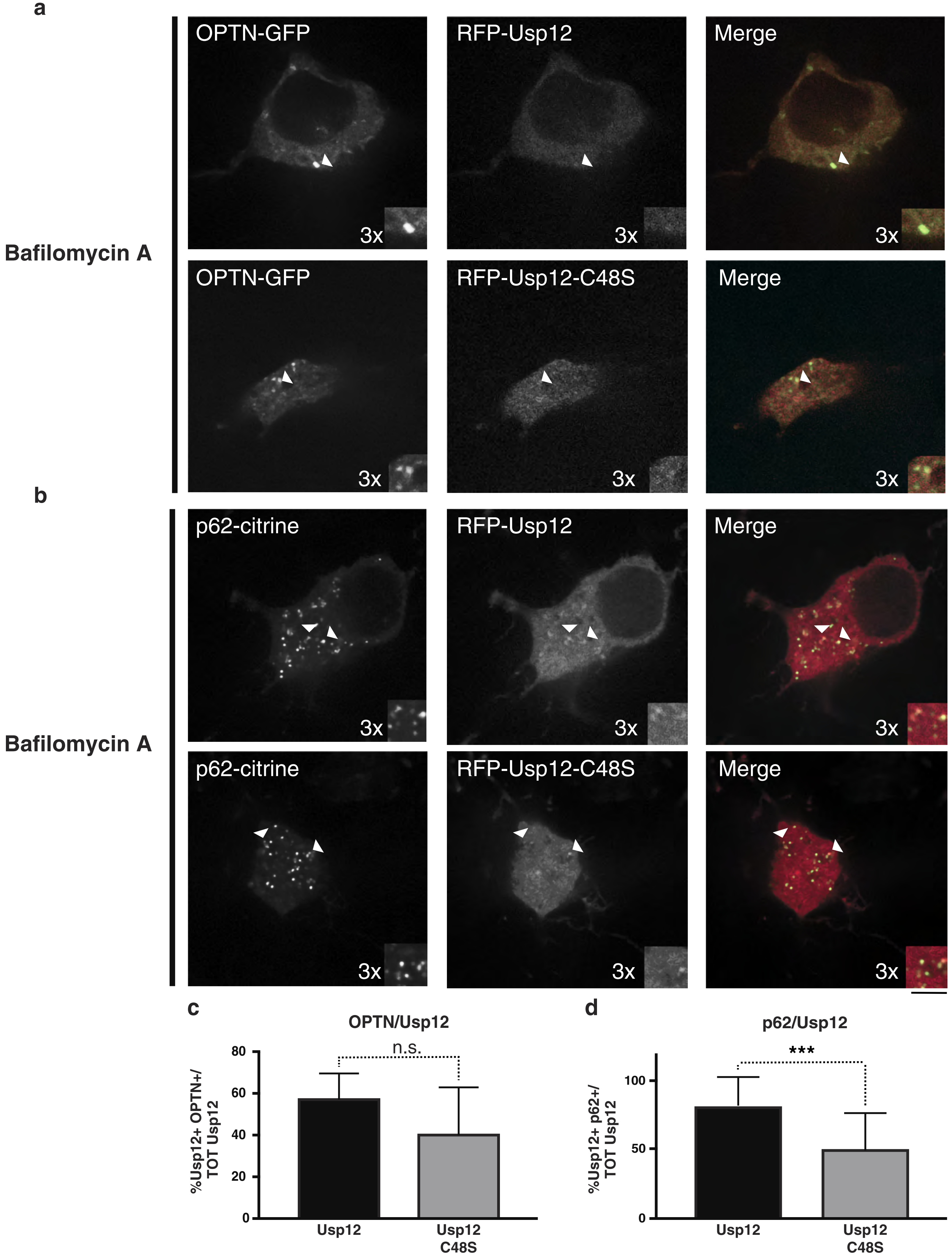


**Supplementary Figure 8.** Usp12 localizes to autophagy receptors. (a,b ) Representative images of RFP-Usp12 co-transfected with either Optineurin (Optn)-GFP (a) or p62-citrine (b) = in primary neurons. Images were captured in fixed cells approximately 48 h after transfection and after 3 h incubation with Bafilomycin A. Scale bar=10 µm. (c-d) quantification of the Usp12 puncta that co-localize with either OPTN or p62, at least 10-15 cells per condition were quantified. Student's t-test was used to compare groups *p<0.01***p<0.0001, n.s.=not significant. Data are representative of three independent experiments. Error bars represent s.e.m..

## Supplementary Figure 9

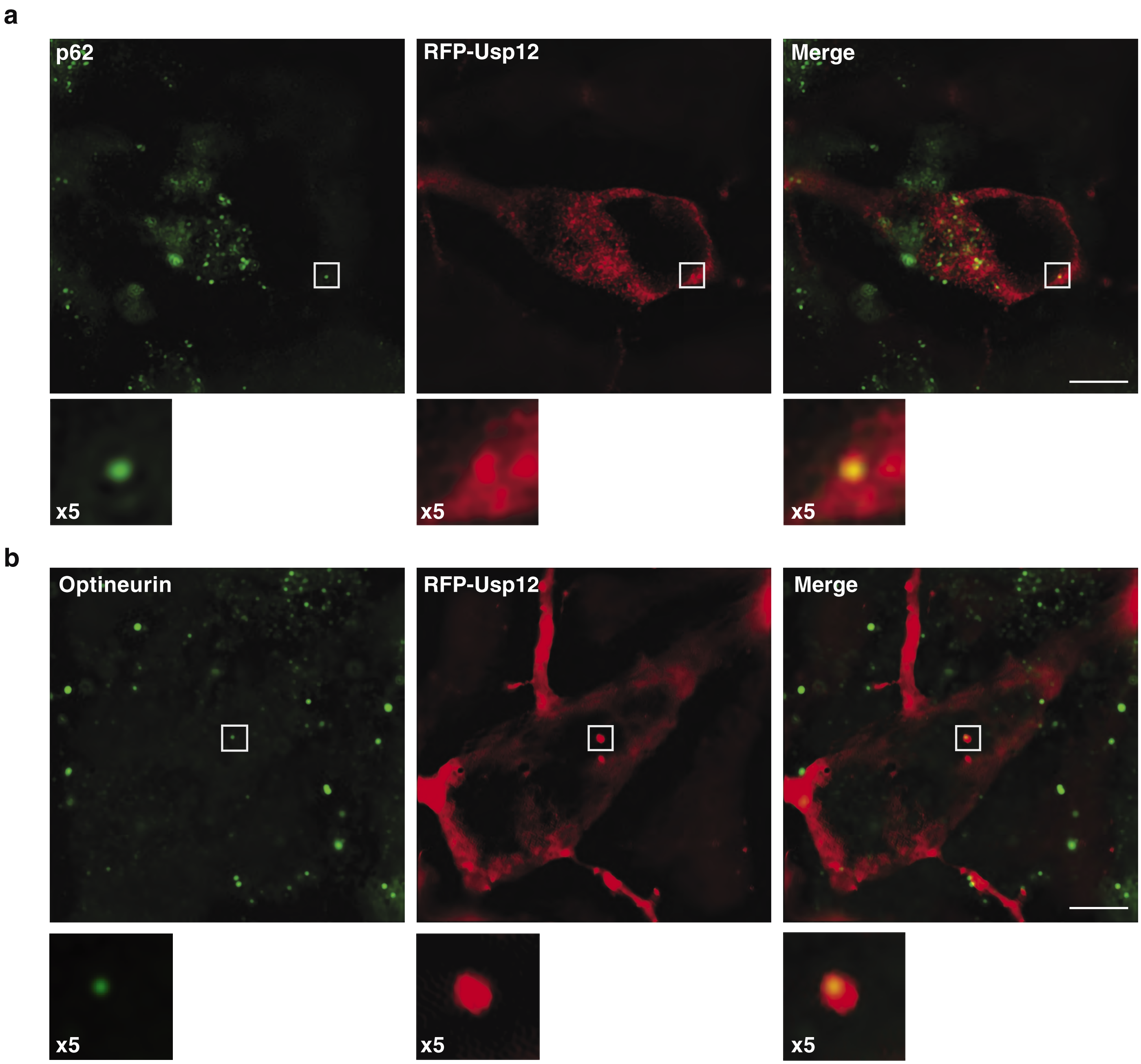


**Supplementary Figure 9.** Representative examples of reconstructed super-resolution images are shown for each pattern. Usp12-mAPPLE expressing cells were incubated with anti-p62 antibody (a) or anti-optineurin antibody (b). Colocalized puncta are represented in the insets. Scale bar: 5µm

## Supplementary Figure 10

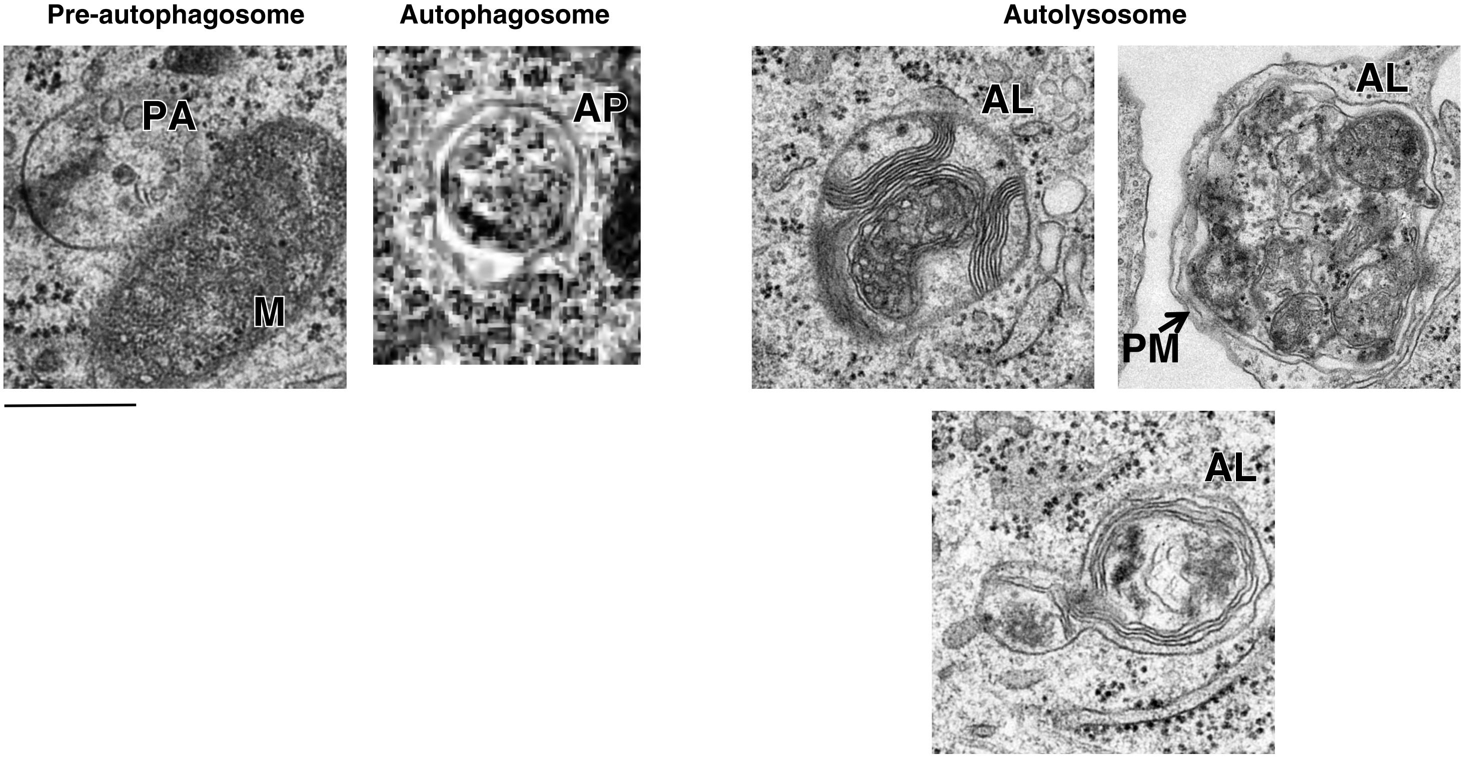


**Supplementary Figure 10.** Examples of autophagic structures included in analysis. PA=pre-autophagosome, M=mitochondria, AP=autophagosome, AL=autolysosome, PM=plasma membrane. Scale bar= 500 nm

# Supplementary Figure 11

Figure 3g

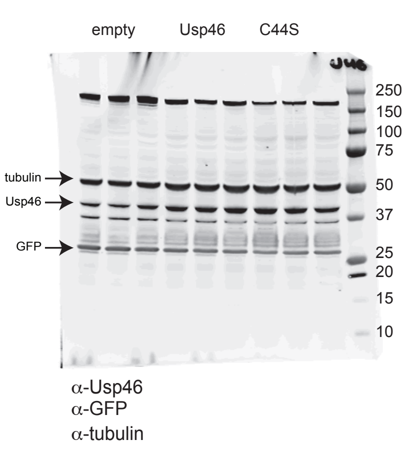


Figure 4d

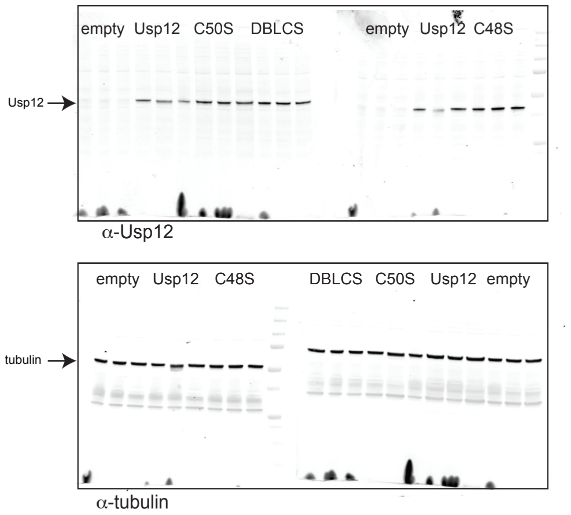


Figure 5e

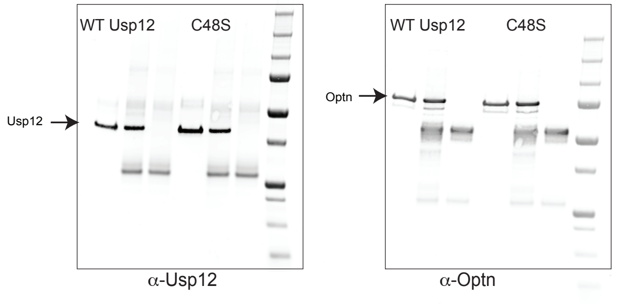


Figure 5f

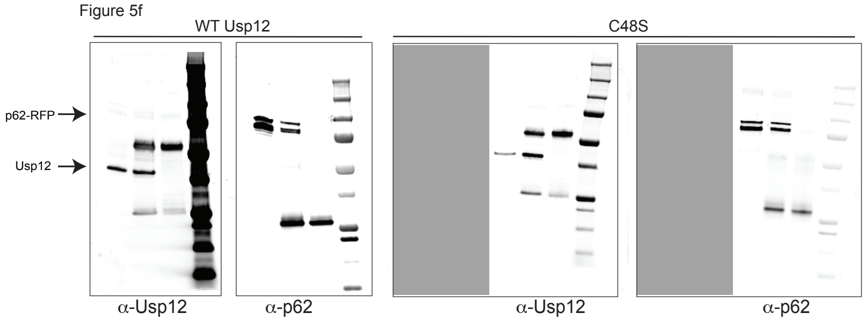


Supplementary Figure 1c

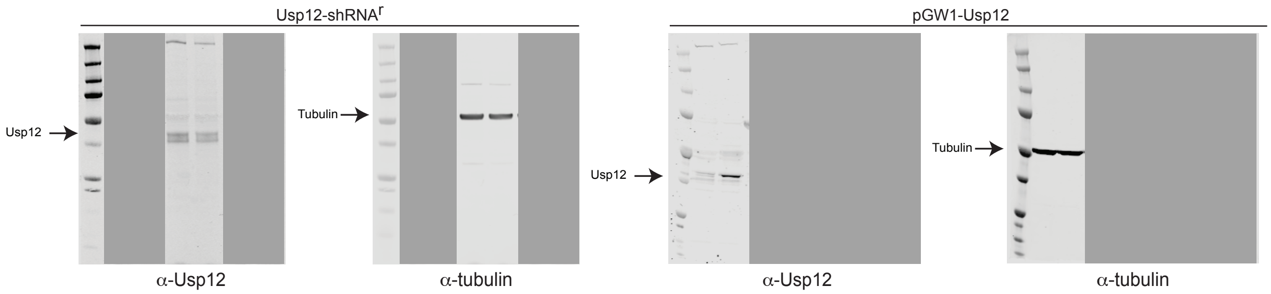


Data not used in this manuscript

**Supplementary Figure 11.** Full immunoblots. Molecular mass marker labeled in full blot for Figure 3f, and is the same marker in all other blots.

**Supplementary Table 1.** Cox proportional hazards analysis of the effects of Usp12 knockdown on survival of rodent primary neurons

| | HR | HR 95% CI | *p* value | n |
|---|---|---|---|---|
| HTT-N586$^{Q17}$ | — | — | — | 258 |
| nt-siRNA | | | | |
| Usp12-siRNA$^{r}$ | 1.15 | 0.85–1.56 | 0.34 | 506 |

HR, hazard ratio; n, number of neurons; CI, confidence interval

**Supplementary Table 2.** Cox proportional hazards analysis of the effects of Usp12 knockdown or overexpression on survival of rodent primary neurons.

| | HR | 95% CI | *p* value | n |
|---|---|---|---|---|
| HTT-N586$^{Q17}$ | — | — | — | 240 |
| mHTT-N586$^{Q138}$ | | | | |
| nt-siRNA + control vector | 1.45 | 1.12–1.89 | 0.0054 | 196 |
| Usp12-siRNA$^{r}$ + control vector | 2.10 | 1.67–2.66 | 6.2e-10 | 260 |
| Usp12-siRNA$^{r}$ + Usp12 | 1.33 | 1.04–1.71 | 0.0241 | 238 |
| Usp12-siRNA$^{r}$ + Usp12 + C48S | 1.14 | 0.84–1.54 | 0.3961 | 136 |

HR, hazard ratio; n, number of neurons; CI, confidence interval

**Supplementary Table 3.** Cox proportional hazards analysis of Usp12 effect on survival of patient-derived iPSC-differentiated neurons.

| | HR | HR 95% CI | *p* value | n |
|---|---|---|---|---|
| Control + Control vector | Reference | — | — | 129 |
| Control + Usp12 | 0.97 | 0.62–1.50 | 0.8775 | 128 |
| HDQ109 + Control vector | 1.49 | 1.02–2.19 | 0.0394 | 179 |
| HDQ109 + Usp12 | 0.94 | 0.63–1.41 | 0.7759 | 201 |
| Control + Control vector | 0.67 | 0.46–0.98 | 0.03942 | 129 |
| Control + Usp12 | 0.65 | 0.44–0.95 | 0.02774 | 128 |
| HDQ109 + Control vector | Reference | — | — | 179 |
| HDQ109 + Usp12 | 0.63 | 0.45–0.89 | 0.00856 | 201 |

HR, hazard ratio; n, number of neurons; CI, confidence interval

**Supplementary Table 4.** Cox proportional hazards analysis of Usp12 effect on survival of neurons expressing toxic neurodegenerative disease-related proteins

| **Usp12 overexpression (ALS model)** | **HR** | **HR 95% CI** | ***p* value** | **n** |
|---|---|---|---|---|
| GFP+ Control vector | Reference | — | — | 194 |
| GFP+ Usp12 | 1.04 | 0.80–1.35 | 0.779 | 239 |
| TDP-43 + Control vector | 1.90 | 1.48–2.42 | 3.30e-07 | 235 |
| TDP-43+ Usp12 | 2.27 | 1.78–2.90 | 3.04e–11 | 243 |
| GFP + Control vector | Reference | — | — | 151 |
| GFP + Usp12 | 0.89 | 0.65–1.23 | 0.482 | 150 |
| TDP-43$^{A315T}$ + Control vector | 1.97 | 1.49–2.60 | 1.81e–06 | 168 |
| TDP-43$^{A315T}$ + Usp12 | 2.39 | 1.82–3.13 | 3.75e–10 | 186 |
| **Usp12 overexpression (PD model)** | | | | |
| GFP + Control vector | Reference | — | — | 488 |
| GFP + Usp12 | 1.07 | 0.90–1.27 | 0.417 | 446 |
| $\alpha$-synuclein + Control vector | 1.44 | 1.24–1.69 | 3.45e–06 | 534 |
| $\alpha$-synuclein + Usp12 | 1.63 | 1.39–1.90 | 1.41e–09 | 483 |

HR, hazard ratio; n, number of neurons; CI, confidence interval

**Supplementary Table 5.** Cox proportional hazards analysis of differential effects of Usp46 on neuron survival

| **Usp46 overexpression** | **HR** | **HR 95% CI** | ***p* value** | **n** |
|---|---|---|---|---|
| HTT-N586$^{Q17}$ + Control vector | Reference | — | — | 382 |
| HTT-N586$^{Q17}$ + Usp46 | 1.78 | 1.46–2.17 | 1.67e–08 | 394 |
| mHTT-N586$^{Q138}$ + Control vector | 1.85 | 1.52–2.25 | 1.10e–09 | 411 |
| mHTT-N586$^{Q138}$ + Usp46 | 1.83 | 1.50–2.23 | 2.89e–09 | 386 |
| **WT and mutant Usp46 overexpression** | | | | |
| HTT-N586$^{Q17}$ + Control vector | Reference | — | — | 160 |
| HTT-N586$^{Q17}$ + Usp46 | 2.25 | 1.63–3.11 | 7.76e–07 | 146 |
| HTT-N586$^{Q17}$ + Usp46-C44S | 1.26 | 0.83–1.92 | 0.276 | 74 |
| mHTT-N586$^{Q138}$ + Control vector | Reference | — | — | 192 |
| mHTT-N586$^{Q138}$ + Usp46 | 0.98 | 0.74–1.31 | 0.912 | 137 |
| mHTT-N586$^{Q138}$ + Usp46-C44S | 0.97 | 0.73–1.28 | 0.832 | 149 |
| **WT and mutant Usp12/Usp46 overexpression** | | | | |
| GFP + Control vector | Reference | — | — | 178 |
| GFP + Usp46 | 1.84 | 1.40–2.43 | 1.6e–05 | 173 |
| GFP + Usp46-C44S | 1.01 | 0.74–1.37 | 0.947 | 165 |
| GFP + Usp12 | 0.97 | 0.71–1.33 | 0.857 | 158 |
| GFP + Usp12-C48S | 1.18 | 0.87–1.64 | 0.317 | 119 |

HR, hazard ratio; n, number of neurons; CI, confidence interval

**Supplementary Table 6.** Cox proportional hazards analysis of the effects of Wdr20 and Wdr48 knockdown in survival of mHTT neurons

| | HR | 95% CI | *p* value | n |
|---|---|---|---|---|
| mHTT-N586$^{Q138}$ | | | | |
| Control vector + nt-siRNA | Reference | — | — | 551 |
| Usp12-C48S + nt-siRNA | 0.69 | 0.59–0.81 | 4.52e–06 | 292 |
| Usp12-C48S + Wdr48-siRNA | 0.65 | 0.56–0.75 | 6.28e–09 | 406 |
| Usp12-C48S + Wdr20-siRNA | 0.67 | 0.58–0.78 | 7.72e–08 | 391 |
| Usp12-C48S + Wdr48-siRNA + Wdr20-siRNA | 0.59 | 0.50–0.69 | 5.84e–09 | 280 |

HR, hazard ratio; n, number of neurons; CI, confidence interval.

**Supplementary Table 7.** Effect of Usp12 on mHTT-N586$^{Q138}$–related risk factors, expression level and IB formation, on neuronal survival.

| mHTT-N586$^{Q138}$+ | Covariate | HR | 95% CI | *p* value |
|---|---|---|---|---|
| Empty vector (n=376) | mHTT-N586$^{Q138}$ fluorescence (a.u.) | 6.30776 | 2.802–14.200 | 8.7e–06 *** |
| | IB formation | 0.15909 | 0.052–0.487 | 0.00127 ** |
| | IB formation:time | 1.03065 | 1.013–1.048 | 0.00043 *** |
| Usp12-WT (n=293) | mHTT-N586$^{Q138}$ fluorescence (a.u.) | 1.4579 | 1.2888–1.649 | 2.1e–09 *** |
| | IB formation | 0.0932 | 0.0166–0.522 | 0.0069 ** |
| | IB formation:time | 1.0324 | 1.0093–1.056 | 0.0057 ** |
| Usp12-C48S (n=332) | mHTT-N586$^{Q138}$ fluorescence (a.u.) | 8.95395 | 4.9181–16.302 | 7.5e–13 *** |
| | IB formation | 0.14141 | 0.0401–0.499 | 0.0024 ** |
| | IB formation:time | 1.02791 | 1.0094–1.047 | 0.0030 ** |

IB, inclusion body; n, number of neurons; HR, hazard ratio; CI, confidence interval; a.u., arbitrary unit.

**Supplementary Table 8.** Competing risks regression analysis of the effects of Usp12 on risk of mHTT-N586$^{Q138}$IB formation.

| mHTT-N586$^{Q138}$+ | HR | 95% CI | *p* value |
|---|---|---|---|
| Empty vector (n=376) | Reference | — | — |
| Usp12-WT (n=293) | 0.918 | 0.714–1.18 | 0.58 |
| Usp12-C48S (n=332) | 1.034 | 0.801–1.33 | 0.83 |

IB, inclusion body; n, number of neurons; HR, hazard ratio; CI, confidence interval

**Supplementary Table 9.** Cox proportional hazards analysis of the effects of Usp12 on mHTT toxicity during autophagy inhibition by Atg7 knockdown

| | | HR | 95% CI | *p* value | n |
|---|---|---|---|---|---|
| mHTT-N586$^{Q138}$ + | | | | | |
| nt-shRNA | Control | Reference | — | — | 504 |
| | Usp12 | 0.67 | 0.537–0.827 | 2.4e–04 | 411 |
| | Usp12-C48S | 0.52 | 0.402–0.675 | 7.7e–07 | 302 |
| Atg7-shRNA | Control | Reference | — | — | 406 |
| | Usp12 | 1.01 | 0.847–1.20 | 0.92 | 446 |
| | Usp12-C48S | 0.97 | 0.810–1.16 | 0.73 | 415 |

HR, hazard ratio; n, number of neurons; CI, confidence interval.
